# Controlling Particle Morphology and Pore Size in the Synthesis of Ordered Mesoporous Materials

**DOI:** 10.3390/molecules25214909

**Published:** 2020-10-23

**Authors:** Yaregal Awoke, Yonas Chebude, Isabel Díaz

**Affiliations:** 1 Instituto de Catálisis y Petroleoquímica, CSIC, C/Marie Curie 2, 28049 Madrid, Spain; yaregal0918@yahoo.com; 2 Department of Chemistry, Arat Kilo Campus, Addis Ababa University, Addis Ababa 1230, Ethiopia; yonasdb1@yahoo.com

**Keywords:** SBA-15, PMO, particle morphology, pore size, surface hydrophobicity

## Abstract

Ordered mesoporous materials have attracted considerable attention due to their potential applications in catalysis, adsorption, and separation technologies, as well as biomedical applications. In the present manuscript, we aim at a rational design to obtain the desired surface functionality (Ti and/or hydrophobic groups) while obtaining short channels (short diffusion paths) and large pore size (>10 nm). Santa Barbara Amorphous material SBA-15 and periodic mesoporous organosilica PMO materials are synthesized using Pluronic PE 10400 (P104) surfactant under mild acidic conditions to obtain hexagonal platelet-like particles with very short mesochannels (300–450 nm). The use of expanders, such as 1, 3, 5-trimethylbenzene (TMB) and 1, 3, 5-triisopropylbenzene (TIPB) were tested in order to increase the pore size. TMB yielded in the formation of vesicles in all the syntheses attempted, whereas P104 combined with TIPB resulted both in expanded (E) E-SBA-15 and E-PMO with 12.3 nm pore size short channel particles in both cases. Furthermore, the synthesis method was expanded to the incorporation of small amount of Ti via co-condensation method using titanocene as titanium source. As a result, Ti-E-SBA-15 was obtained with 15.5 nm pore size and isolated Ti-sites maintaining platelet hexagonal morphology. Ti-PMO was obtained with 7.8 nm and short channels, although the pore size under the tried synthesis conditions could not be expanded further without losing the structural ordering.

## 1. Introduction

The synthesis, characterization, and application of novel porous materials have been strongly encouraged due to their wide range of applications in adsorption, separation, catalysis, and biomedical field. Since the breakthrough achieved by the M41S family, reported by Mobil’s researchers in 1992 [1], ordered mesoporous silica have attracted an extraordinary scientific interest in an interdisciplinary set of fields depending on the desired application. Due to the limited pore size of M41S materials, a new class of well-ordered mesoporous silica materials was synthesized at the University of California, being denoted as Santa Barbara Amorphous materials (SBA-15) in 1998 [2], which have relatively larger pore sizes and pore wall thicknesses as compared with the M41S family. SBA-15 materials are silica structures that present cylindrical pores of uniform diameter placed in hexagonal arrangement. Silicate mesoporous materials with high surface areas, combined with large and uniform pore sizes, have acquired great scientific interest in the use as hosts to support catalytic active sites [3] or to confine guest molecules, such as enzymes [4]. Periodic mesoporous organosilicas (PMO) with highly ordered pore structures and uniformly distributed organic groups inside of siliceous framework provided with a new range of surface chemistry in ordered mesoporous materials [5]. PMOs have been synthesized via the hydrolysis and condensation of bridged organosilsesquioxane species ((RO)_3_Si-R-Si(OR)_3_) under basic or acidic conditions in the presence of various structure-directing agents [6].

Non-ionic surfactant Pluronic P123 (EO_20_PO_70_EO_20_) is commonly used in the synthesis of SBA-15 and PMO. The resulting morphology in these syntheses is usually large fibers, in the orders of hundreds of microns. The lengthy channels and potential pore blockage along the channels are the main concerns when applying SBA-15 and PMO type ordered mesoporous materials in the sorption and catalysis of bulky molecules. The pore accessibility and molecular diffusion path in such types of materials can be improved by shortening the mesopore channel length [7] or enlarging the mesopore diameter [8].

In this study, new synthetic strategies were adopted to prepare SBA-15 and PMO with short channels, hexagonal platelet-like morphology, and large pore size (>10 nm). This would improve the accessibility of the potential active sites and the diffusion and adsorption rate of the reactants and products [9]. Non-ionic Pluronic PE 10400 (P104, EO_27_PO_61_EO_27_) surfactant has been selected as structure-directing agent in order to control the particle morphology and textural properties [10]. The nature of the structure directing agent, the synthesis temperature, and the addition of other synthesis agents, such as inorganic salts and swelling agents, are key parameters that should be considered to control the properties of the final material. Inorganic salts improve the degree of ordering of PMO materials by the dehydration of the ethylene oxide (EO) units of the surfactant, which decreases their hydrophilicity and increases the hydrophobicity of the propylene oxide (PO) moieties. Thus, the interaction between the positively charged polyethylene oxide (PEO) groups and the relatively hydrophobic positively charged organosilane species is favored. The use of expanders is known in the conventional synthesis of SBA-15 with Pluronic P123 [11], although it has not been thoroughly tried with Pluronic PE 10400, with slightly more hydrophilic character that usually forms shorter micelles, which promotes SBA-15 growing preferentially in the *ab* plane [12]. Furthermore, the use of Pluronic PE 10400 has not been attempted to prepare PMO with hexagonal platelet-like morphology let alone, to prepare expanded pore-PMO materials.

In this work, we introduce the morphology control using Pluronic P104 to the synthesis of PMO, and develop a method to obtain large pore, or expanded, E-SBA-15 and E-PMO in the synthesis conditions that produce hexagonal platelet-like morphology. Furthermore, in the course of our work to produce short diffusion paths in catalysis, we extended to a facile method to synthesize platelet-like Ti-SBA-15 and Ti-PMO particles with good textural properties and short channel cylindrical mesopores in the presence of small amount of titanocene dichloride. Based on early reports on the use of titanocene as the optimum source for the incorporation of isolated active oxidative sites [13], we expanded the synthetic approach to the use of Pluronic PE 10400 and expanders, such as TIPB obtaining, expanded Ti-surface modified SBA-15 and PMO.

## 2. Results

### 2.1. Characterization of SBA-15 and Ti-SBA-15 Ordered Mesoporous Materials

The obtained samples were characterized by low angle powder X-ray diffraction to evaluate the pore arrangement, scanning and transmission electron microscopy to evaluate the morphology and internal structure of the particles, and N_2_ adsorption/desorption isotherm, to obtain the textural properties of the supports.

**Figure 1:**
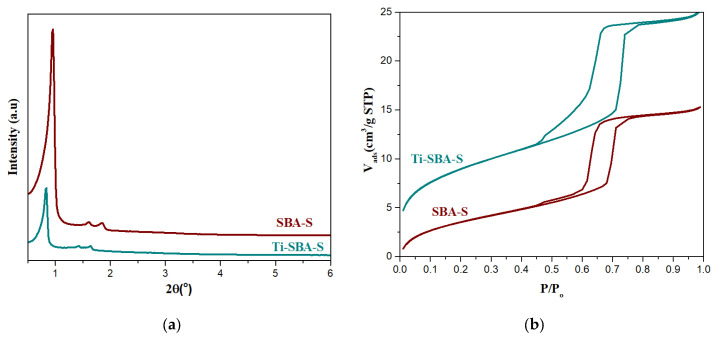
(**a**) Low angle XRD pattern; (**b**) N_2_ adsorption-desorption isotherms of “short” SBA-15 materials SBA-S and Ti-SBA-S.

Figure 1a shows the low angle X-ray diffraction patterns for the materials prepared with Pluronic P104, labeled with “S” as short, SBA-S and Ti-SBA-S, aiming at short channels. Both SBA-S and Ti-SBA-S materials show a typical XRD pattern of an ordered hexagonal network arrangement of mesopores. The presence of one very intense diffraction peak, d_100_, and two weak peaks d_110_ and d_200_ in both materials characterize a well-defined 2D hexagonal symmetry mesoporous structure belonging to the *p*6*mm* space group.

Interestingly, the incorporation of small amount of titanocene does not alter the formation of a highly ordered hexagonal structure. Ti-SBA-S is obtained with 5% of titanocene in the synthesis gel provoking the co-condensation of Ti-cyclopentadienyl (Cp) species to condense with the silicate at the pore surface. As a result, this sample shows slightly larger unit cell (12.1 nm) than the pure silica (10.6 nm) (Figure 1a and Table 1). This larger unit cell parameter may be indicating that there is an efficient interaction of cyclopentadienyl with the core of the micelle causing the pore size and unit cell dimension increment. The N_2_ adsorption-desorption isotherms of calcined SBA-S and Ti-SBA-S materials are shown in Figure 1b and their textural properties are summarized in Table 1. The adsorption/desorption isotherm of both materials exhibit the classic type IV adsorption isotherms, according to the IUPAC classification [14], with an H1-type hysteresis loop, which is characteristic of mesoporous materials with uniformly distributed cylindrical channels. The adsorption and desorption branches are located at high relative pressure (P/P_o_ between 0.5–0.8) characteristic of mesoporous materials. The adsorption branch of Ti-SBA-S material is shifted slightly toward higher P/P_o_, which suggests an increase of the pore size. This pore size difference is also confirmed by DFT (Density functional theory) calculations, the pore size of SBA-S and Ti-SBA-S materials are 9 and 10 nm, respectively. The pore size, surface area and pore volume of Ti-SBA-S material is slightly higher than the SBA-S material.

**Figure 2:**
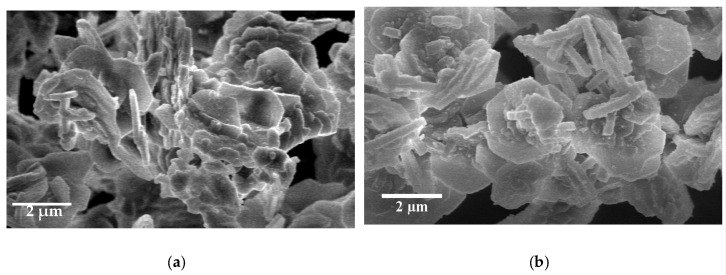
Scanning electron microscopy micrographs of: (**a**) SBA-S; (**b**) Ti-SBA-S materials.

The particle morphology of both SBA-S and Ti-SBA-S materials was characterized by scanning electron microscopy (SEM) to confirm whether our objective has been achieved or not. SBA-S material is composed of platelet particles, with diameters of 3.2 µm, while the materials synthesized in the presence of titanocene dichloride, Ti-SBA-S, have hexagonal platelets-like particle morphology with diameter of 2 μm (Figure 2). Although both materials have short channels and plates-like particle morphology, hexagonal particles with smaller diameter is shown in the Ti-SBA-S material. This different growth rate may be related to the milder acidic conditions employed in the Ti-synthesis, although it could also be due to the delay in the initial condensations step due to the presence of titanocene dichloride.

**Figure 3:**
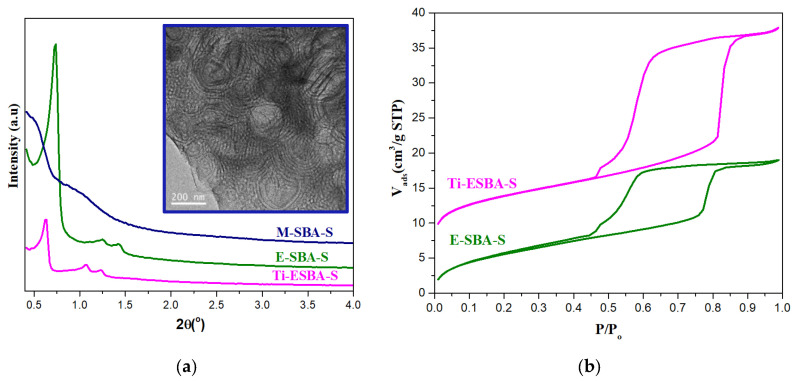
(**a**) Low angle XRD patterns of expanded samples: M-SBA-S using TMB, and E-SBA-S and Ti-E-SBA-S using TIPB. Inset corresponds to the TEM image of M-SBA-S showing vesicles; (**b**) N_2_ adsorption-desorption isotherms of E-SBA-S and Ti-E-SBA-S materials.

In order to facilitate the adsorption and diffusion rate of bulky molecules, we tried to prepare materials with larger pore diameter than the standard pore size materials. Several experimental methods were attempted, only the most relevant ones are highlighted here. Initially, we tried to expand the pore size of SBA-S using 1, 3, 5-trimethylbenzene (TMB) as a pore expander (sample code M-SBA-S). Figure 3a shows the low angle X-ray diffraction patterns for the synthesized mesoporous silica materials with pore expanders. All XRD peaks of M-SBA-S disappear, suggesting that the addition of (TMB) leads to the loss of the hexagonal arrangement of pores. This may be due to uncontrolled interaction with the triblock copolymers that causes disruption of the micellar phase of P104 producing less ordered mesocellular foam material with a wider pore size [15]. TEM image in the inset of Figure 3a corroborates the ringed multi-lamellar silica vesicles with no open pores and no hexagonal arrangements. This result is less pronounced when using P123, yet it has been discussed in the literature as a result of the pore expansion [16,17]. Therefore, we tried to expand the pore size of both types of materials by using another type of pore expander, 1, 3, 5-triisopropylbenzene (TIPB), which has relatively large size and more hydrophobic group. This characteristic may help the expander to selectively interact with the core of the micelle. Figure 3a shows the low angle X-ray diffraction patterns for the synthesized expanded SBA-15 materials (labeled with “E”) for both silica E-SBA-S and with a small amount of titanocene in the synthesis gel, Ti-E-SBA-S. It can be observed that both E-SBA-S and Ti-E-SBA-S materials exhibit XRD patterns with one very intense diffraction peak (100), and two weak peaks (110) and (200), which are characteristic of 2-dimensional hexagonal *p*6*mm* structure with excellent uniformity. The XRD peaks of both materials were shifted towards lower 2θ value (larger a_o_, see Table 1) when compared to their SBA-S and Ti-SBA-S parent materials. This confirms that TIPB increases the unit cell dimension without affecting the pore arrangement of the material. These materials were further characterized by N_2_ adsorption-desorption isotherm in order to confirm the pore size difference with the parent material.

The N_2_ adsorption-desorption isotherms of the calcined E-SBA-S and Ti-E-SBA-S materials are given in Figure 3b and their textural properties are summarized in Table 1. Both materials exhibit the classic type IV adsorption isotherms, with an H1-type hysteresis loop which is characteristic of mesoporous materials with cylindrical channels. The adsorption and desorption branches of these materials are located at higher relative pressure and the hysteresis loop shifted slightly towards higher relative pressure (P/P_o_ = 0.9) when compared to their parent ones, which suggests an increase of the pore size. However, the presence of two distinctive steps in the desorption branch of the isotherm may indicate the presence of partial blockage of some mesopores or presence of bottlenecks. The pore size obtained from the adsorption branch using DFT cylindrical model yields successfully larger pore sizes in both cases, as large as 12.3 nm for pure silica sample and 15.5 nm for the Ti-modified SBA-15 materials, which are rather large compared to those reported in the literature, at least for Ti-SBA-15 [18].

**Figure 4:**
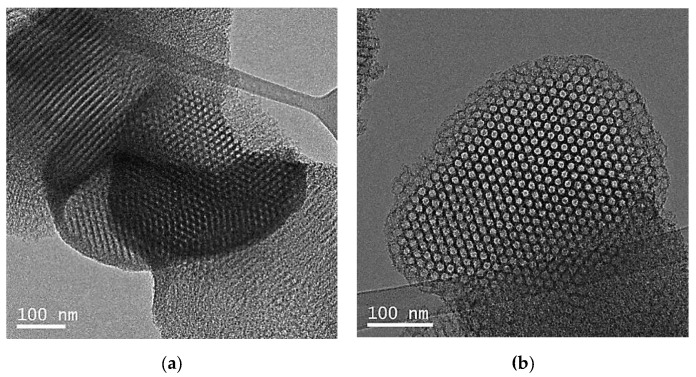
TEM images of: (**a**) E-SBA-S and **(b)** Ti-E-SBA-S materials.

In order to understand the presence of this small second step, these samples were further studied by TEM. Figure 4 shows the TEM images of both E-SBA-S and Ti-E-SBA-S depicting the well-ordered mesoporous structures in both materials. The presence of very thick layers surrounding the outer surface of the particles in E-SBA-S may explain the presence of partially blocked pore mouths (lack of pore openings); however, no blockage was observed in Ti-E-SBA-S material, where the channels and the pore mouths are accessible [9].

**Figure 5:**
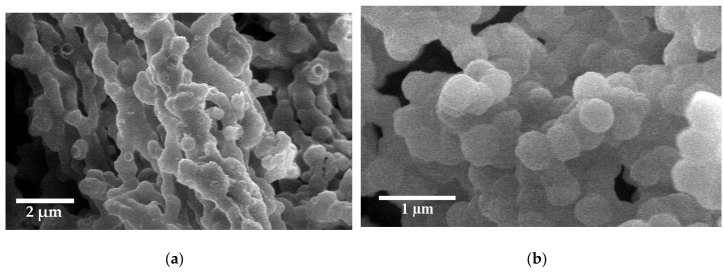
Scanning electron microscopy micrographs of: (**a**) E-SBA-S; (**b**) Ti-E-SBA-S materials.

The particle morphology of both E-SBA-S and Ti-E-SBA-S materials were analyzed by SEM (Figure 5). E-SBA-S seems to form fibers but the fibers being formed by aggregates Figure 5a. The particle morphology of E-SBA-S is affected by the addition of the TIPB expander probably due to the different particle growth mechanism when compared with the parent SBA-S material. In the Ti-E-SBA-S material discrete particles are formed, attached short end to short end Figure 5b. When the synthesis is conducted in the presence of expander, the size of the crystals is much smaller, 0.5 μm particle diameter, having the shape of small cylinders.

### 2.2. Characterization of PMO and Ti-PMO Hybrid Ordered Mesoporous Materials

**Figure 6:**
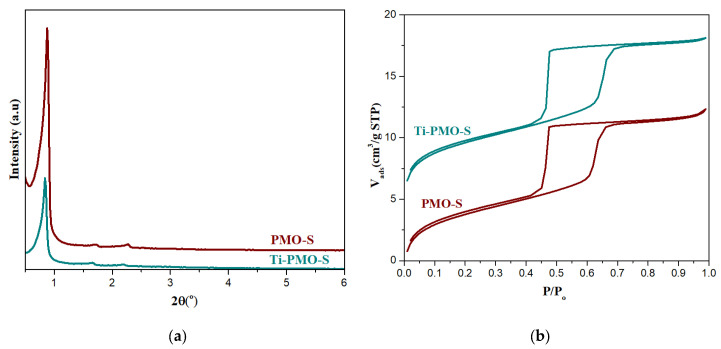
(**a**) Low angle XRD patterns; (**b**) N_2_ adsorption-desorption isotherms of PMO-S and Ti-PMO-S materials.

In order to tune the adsorption capacity and rate of diffusion of organic reagents the synthesis of materials with hydrophobic group, periodic mesoporous organosilica, with short particle morphology and large pore size was aimed. The synthesis condition of PMOs is carried out at mild acidic conditions. Figure 6a shows the low angle X-ray diffraction patterns of PMO-S and Ti-PMO-S materials. The presence of one very intense peak (100) and two weak peaks (110) and (200) in both materials indicates that both are ordered materials with the hexagonal arrangement of mesopores belonging to *p*6*mm* symmetry. The presence of small amount of titanocene in the synthesis gel does not seem to alter the structure directing effect of P104 surfactant even when the silica source is a bridged one. Ti-PMO-S material has slightly larger unit cell dimensions than PMO, 12 and 11.6 nm, respectively (see Table 1).

**Figure 7:**
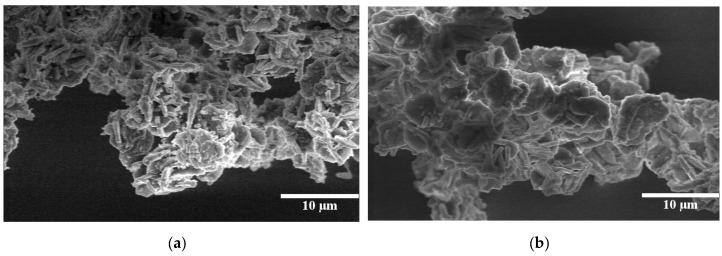
Scanning electron micrographs of: (**a**) PMO-S; (**b**) Ti-PMO-S materials.

These materials were characterized by N_2_ adsorption/desorption isotherms to compare their textural properties. Figure 6b shows N_2_ isotherms for the calcined hybrid organosilica materials. The isotherms of both materials are type IV, with H1 type hysteresis loop at high relative pressure, which is characteristic of mesoporous materials with uniform size cylindrical pores. The DFT pore size is the same in both materials, close to 8 nm, which is relatively larger when compared with the pore size of Ti-PMO material reported in the literature, which is close to 6 nm, [19]. The desired effect of morphology modification was observed by scanning electron microscopy. SEM images of PMO-S and Ti-PMO-S are shown in Figure 7, showing platelets morphology with short channels. Therefore, from SEM, we can conclude that the particle morphologies of PMO materials can be controlled by using Pluronic P104 as a surfactant, and particle dimension of 3.2 µm diameter by 550 nm length is obtained. This channel dimension is roughly similar with the SBA-S material, which has been synthesized by using the same type of surfactant but different type of silica source.

**Figure 8:**
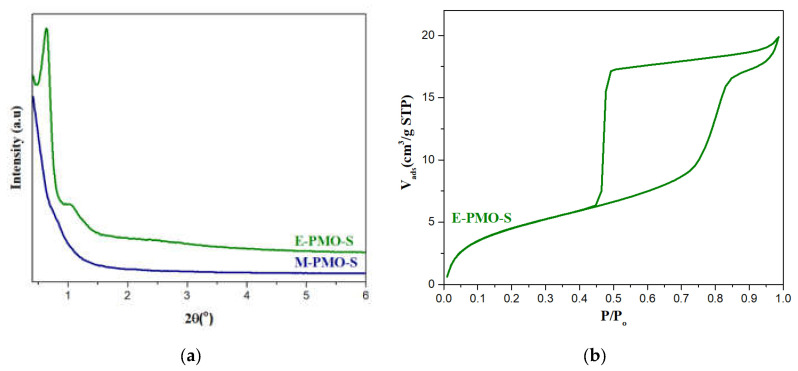
(**a**) Low angle XRD patterns; (**b**) N_2_ adsorption-desorption isotherm of E-PMO-S.

Different attempts were made to synthesize large pore size PMO materials using 1, 3, 5-trimethylbenzene (TMB) as a pore expander (M-PMO-S). However, all the XRD peaks disappear indicating that the M-PMO-S material completely loses its structure due to the effect of TMB similarly as M-SBA-S sample. Thus, we tried to expand the pore size of the PMO materials using TIPB as a pore expander. Figure 8a shows the low angle X-ray diffraction patterns for an expanded E-PMO material prepared with TIPB using P104 as surfactant. The presence of one strong peak (100) and two less resolved peaks (110) and (200) of E-PMO-S corroborates that the structure is less ordered, yet the main peak in this material is shifted towards the lower 2θ, which indicates that the unit cell dimension increases due to the effect of the micelle expander.

**Figure 9:**
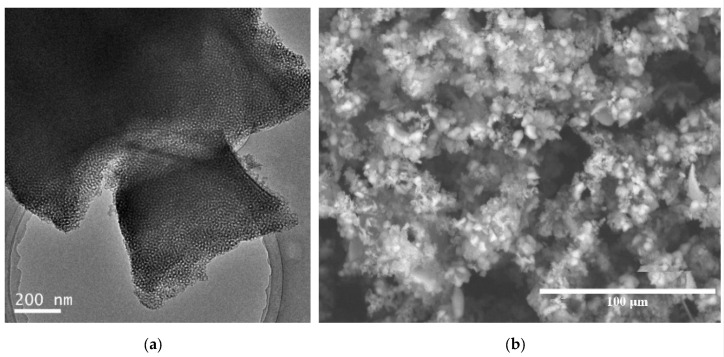
Structure and morphology of large pore size E-PMO-S material (**a**) TEM and (**b**) SEM images.

Figure 8b shows the N_2_ adsorption–desorption isotherm for E-PMO-S material and the textural properties are given in Table 1. The isotherm obtained in E-PMO-S sample is type IV isotherm with H1 type hysteresis loop indicating uniform pore size distribution. However, the adsorption branch of the isotherm is not as sharp as it should be for a highly ordered OMM, probably due to the presence of bottlenecks causing delays in the diffusion of the gas. Nevertheless, the pore size according to DFT calculations has successfully been expanded to 12.3 nm, more than 5 nm larger than the parent material PMO-S (7.1 nm). TEM observations confirmed that the mesoporous structure in this material is indeed not hexagonally packed, rather, it is formed by disordered arrangement with wormlike mesochannels [20]. The particle morphology of E-PMO-S material is given in Figure 9b, showing agglomerates of shapeless small particles.

## 3. Discussion

SBA-15 and PMO materials are synthesized using nonionic surfactant Pluronic PE104 which has the general formula EO_27_PO_61_EO_27_. Polyethylene oxide (PEO) sections of the surfactant are hydrophilic and responsible for the interaction with the silica source. In acidic media, PEO becomes highly depolymerized and protonated interacting by H-bonds with the protonated silica source. On the other hand, polypropylene oxide (PPO) units are responsible for the hydrophobic core of the micelles. The presence of equivalent amount of hydrophilic PEO and hydrophobic PPO group in the surfactant molecule may assist the formation of short channel and platelet-like particle morphology. This may be due to the side to side interaction among the particles during crystal growth. This fact is clearly supported by Linton et al. [7], which clearly shows the growth mechanism of the particles with plate-like hexagonal prisms morphology by using Pluronic P104 surfactant at high synthesis temperature (50–65 °C) and relatively concentrated acidic condition (1.6 M). Here, we add titanocene dichloride into the synthesis gel at milder acidic condition, and swelling agents, such as TIPB, at lower synthesis temperature.

In this synthesis approach, titanocene dichloride (Cp_2_TiCl_2_) was added before the addition of silica source, during the SBA-15 and PMO synthesis, respectively. In this method of synthesis Ti (IV) ion was incorporated, while maintaining degree of ordering of the material and its particle morphology. The presence of small amount of cyclopentadienyl groups, may facilitate the aggregation of smaller units (primary particles) in the oriented manner, during the formation of ordered mesoporous materials. As compared to their corresponding parent materials, Ti incorporated materials have higher unit cell dimension, larger textural properties (surface area, pore volume, pore diameter and pore wall thickness), perhaps induced by the species from the titanium precursor utilized in the synthesis. Besides, the materials synthesized in the presence of titanocene dichloride Ti-SBA-S and Ti-PMO show a smaller particle size, contributing to the aforementioned better textural properties. This morphological difference may be due to the increase of particle-particle attraction upon the addition of Cp_2_TiCl_2_. This was explained by Linton et al. [12], where the aggregation of primary particles was promoted by adding inorganic salts (NaCl and NaI). Perhaps, in this case, this is due to the effect of both the cation and the anion species present in the synthesis gel.

Ti-E-SBA-S has large pore size and cylindrical shaped particle morphology with diameter 0.5 μm. This result also indicates that the presence of pore expanders in the synthesis gel may facilitate nucleation and hinders particle growth yielding the decrease of the particle. This fact was also observed by Johansson et al. [21] in the synthesis of SBA-15 in the presence of heptane.

In the case of the synthesis of PMO, Pluronic P104 is used here for the first time instead of the more frequently used Pluronic P123 [22], obtaining PMO materials with platelet particle morphology. PMO-S and Ti-PMO-S have similar particle size and pore wall thickness. However, Ti-PMO-S material has slightly higher pore diameter, surface area and pore volume may be due to the modulation of the acidity, or presence of small amount of titanocene dichloride in the synthesis gel that may improve the orientation of the particles [23]. The presence of expander has led to the loss of hexagonal order and, thus, the platelet-like particle morphology.

## 4. Materials and Methods

### 4.1. Materials

Pluronic P104 (PEO_27_PPO_62_PEO_27_) BASF, USA, was used as a structure directing agent. Tetramethoxysilane (TMOS) Alfa Aesar, Germany, ammonium fluoride (NH_4_F), 1, 3, 5-triisopropylbenzene (TIPB), 1, 3, 5-trimethylbenzene (TMB) Alfa Aesar (Germany), 1,2-bis(trimethoxysilyl)ethane (BTMSE, 96%, Sigma Aldrich, St. Louis, MO, USA), and 1,2-bis-(triethoxysilyl)ethane (BTESE, 97%, Merck, NJ, USA were used as a source of silicon and ethane group for PMO synthesis, and titanocene dichloride (Cp_2_TiCl_2_) (Sigma- Aldrich, St. Louis, MO, USA).

### 4.2. Synthesis of SBA-15 and Ti-SBA-15

Pure silica SBA-15 was synthesized using Pluronic PE 10400 (P104, (EO)_27_(PO)_61_(EO)_27_) as a structure directing agent and with tetramethyl orthosilicate (TMOS) as the silica source. Pluronic P104 (2.5 g, 0.423 mmol) was dissolved in 1.6 M aqueous HCl solution (97.5 g). The mixture was stirred at room temperature in a closed Pyrex container until a homogenous clear solution was obtained. Then, the solution was heated at 55 °C for 1 h to homogenize the temperature. TMOS (3.69 mL, 24.79 mmol) was then added to the solution and the resulting mixture was vigorously stirred at 55 °C for 24 h. The final molar composition of the synthesis gel was 1 TMOS: 0.017 P104: 5.25 HCl: 206 H_2_O. Subsequently, the container was transferred to an oven and kept at 80 °C for 24 h under static conditions. The resultant product was filtered, washed thoroughly with absolute ethanol and air-dried at room temperature overnight. Finally, the surfactant template was removed by calcination at 550 °C for 5 h in air (heating ramp rate: 2 °C/min). This material was labeled as SBA-S as in “short”.

The particle morphology of this short channel material was further controlled by adding small amount of titanocene dichloride and ammonium fluoride in the synthesis gel under mild acidic conditions. The final molar composition of the synthesis gel was 1 TMOS: 0.015 P104: 0.05 Cp_2_TiCl_2_: 0.03 NH_4_F: 0.33HCl: 188 H_2_O. Subsequently, the container was transferred to an oven and kept at 60 °C for 48 h under static conditions. This material was labeled as Ti-SBA-S.

To produce expanded pore E-SBA-S, Pluronic PE104 (1.55 g (0.263 mmol)) and NH_4_F (0.016 g (0.432 mmol)) were dissolved in 65.0 mL of 1.1 M aqueous HCl solution at room temperature in a closed Pyrex container with stirring. Then, the micelle expander TIPB (0.8 mL (3.3 mmol)) or TMB (0.46 mL (3.3 mmol)) was added and mixed for 0.5 h, and the calculated amount of TMOS (2.2 mL (14.78 mmol)) was added in under vigorous stirring. The final molar composition of the synthesis gel was 1 TMOS: 0.017 P104: 0.2 TIPB: 0.03 NH_4_F: 4.84 HCl: 236 H_2_O. The white gel obtained was vigorously stirred for 24 h at 15 °C and subsequently heated at 80 °C in the closed container under static conditions for 48 h. The mixture was then filtered, and the solid product was washed with absolute ethanol and dried at room temperature. Finally, the sample was calcined in a furnace for 5 h at 550 °C (heating ramp rate: 2 °C/min). This material was labeled as E-SBA-S.

The same synthesis method with the addition of small amount of titanocene dichloride in the synthesis gel with the molar composition: 1 TMOS: 0.05 Cp_2_TiCl_2_: 0.018 P104: 0.22 TIPB: 0.03 NH_4_F: 5 HCl: 250 H_2_O was labeled as Ti-E-SBA-S.

### 4.3. Synthesis of PMO and Ti-PMO

Ethylene-bridged periodic mesoporous organosilica (PMO) with short channels and platelets-like particle morphology was as follows: Pluronic P104 (3.25 g (0.55 mmol)) was dissolved at room temperature in 126.84 mL of 0.174 M HCl aqueous solution in a flask with slow stirring. Once the surfactant was dissolved, potassium chloride (9.38 g, 125.8 mmol) was added and dissolved in the solution. When the resulting solution was homogenized, it was heated to a constant temperature of 40 °C. Then, 1, 2-bis (triethoxysilyl) ethane (5.83 mL (15.75 mmol)) was added at once with rapid stirring. The final molar composition of the synthesis gel was 1 BTESE: 0.035 P104: 8 KCl: 1.39 HCl: 445 H_2_O. The resulting mixture was stirred at 40 °C for 24 h and then aged at 80 °C under static conditions for 24 h. The solid product was recovered by filtration, washed with ethanol and dried at room temperature for 24 h. Finally, the sample was calcined for 5 h at 350 °C (heating ramp rate: 2 °C/min) and this material was labeled as PMO-S.

The particle morphology and textural properties of this short channel PMO material was further controlled by adding small amount of titanocene dichloride and ammonium fluoride in the synthesis gel under mild acidic conditions. The final molar composition of the synthesis gel was 1 BTESE: 0.033 P104: 0.02 Cp_2_TiCl_2_: 8 KCl: 0.03 NH_4_F: 1.07 HCl: 492 H_2_O. Then, the resultant mixture was stirred at 40 °C for 24 h and hydrothermally aged in the oven at 80 °C under static conditions for 24 h. The solid product was recovered by filtration, washed with ethanol and air-dried overnight. Finally, the surfactant was removed by calcination at 350 °C for 5 h in air (heating ramp rate: 2 °C/min). This material was labeled as Ti-PMO-S.

Pluronic P104 (1.95 g (0.33 mmol)) and NH_4_F (0.01 g (0.27 mmol)) were dissolved at room temperature in 85.24 mL of 0.12 M aqueous HCl solution in a flask with slow stirring. Once the surfactant was dissolved, KCl (5.63 g (75.52 mmol) was added. When the resulting solution was homogenized, it was transferred into an incubator to a constant temperature of 15 °C. Then, TIPB (0.96 mL (3.96 mmol)) was added and stirred for 0.5 h before adding 1,2-bis(trimethoxysilyl)ethane (2.36 mL (9.36 mmol)). The final molar composition of the synthesis gel was 1 BTMSE: 0.035 P104: 0.4 TIPB: 8 KCl: 0.03 NH_4_F: 1.09 HCl: 504 H_2_O. The resulting mixture was stirred at 15 °C for 24 h and then aged at 80 °C under static conditions for 48 h. The solid product was recovered by filtration, washed with ethanol, and dried at room temperature. The surfactant was removed by calcination in a furnace for 5 h at 350 °C (heating ramp rate: 2 °C/min) and this material was labeled as E-PMO-S.

### 4.4. Characterization Techniques

X-ray Diffraction (XRD) Patterns of the samples were obtained with a Philips X’PERT diffractometer using Cu Kα radiation. Nitrogen adsorption-desorption isotherms were measured at −196 °C using the micrometrics ASAP 2420 sorptometer to determine textural properties. All samples were degassed at 350 °C for 16 h. The total pore volume was determined from the amount of nitrogen adsorbed at a relative pressure of 0.97. Pore size distributions were determined from the adsorption branches of isotherms using the DFT model with cylindrical geometry of the pores. Transmission electron microscopy (TEM) micrographs were taken using a JEOL 2100F electron microscope operating at 200 kV. The samples for TEM analysis were prepared by suspending a small amount of solid in ethanol. A drop of this suspension was then dispersed onto a holey carbon film on a copper grid, followed by drying at room temperature. The morphology was evaluated by scanning electron microscopy (SEM) with an FE-SEM FEI Nova NanoSEM 230 microscope with vCD detector using chromium coating.

## 5. Conclusions

The synthesis conditions of mesoporous silica (SBA-15) and organosilica (PMO) molecular sieves could be adjusted using Pluronic P104 to yield hexagonal platelet-like particle morphology, short path lengths with various pore sizes. The use of expanders such as 1, 3, 5-trimethylbenzene (TMB) yielded in the formation of vesicles in all the syntheses attempted, whereas P104 combined with 1, 3, 5-triisopropylbenzene (TIPB) resulted both in expanded E-SBA-15 and E-PMO with 12.3 nm pore size short channel particles in both cases. The addition of small amount (5%) titanocene dichloride in the synthesis gel under mild acidic conditions also allowed obtaining Ti-SBA and Ti-PMO with good structural, morphological and textural properties. Large-pore Ti-E-SBA-15 (15.5 nm) could be obtained combining TIPB and P104 in swollen micelles, via co-condensation of tetramethoxysilane with 5% of Cp_2_TiCl_2_ at low gelling temperature (15 °C). However, the same approach was not successful in the case of Ti-PMO that has not been expanded further without losing the structural ordering.

## Figures and Tables

**Table 1:** Structural parameters of SBA-15 and periodic mesoporous organosilicas (PMO) samples.

Sample	a_o_ (nm)	S_BET_ (m^2^g^−1^)	V (cm^3^g^−1^)	D_DFT_ (nm)
SBA-S	10.6	579	0.65	9
Ti-SBA-S	12.1	896	0.93	10.1
E-SBA-S	13.7	701	0.76	12.3
Ti-E-SBA-S	16.2	822	1.17	15.5
PMO-S	11.6	672	0.57	7.1
Ti-PMO-S	12	706	0.59	7.8
E-PMO-S	15.9	843	0.86	12.3
